# Returning findings within longitudinal cohort studies: the 1958 birth cohort as an exemplar

**DOI:** 10.1186/1742-7622-11-10

**Published:** 2014-08-04

**Authors:** Susan E Wallace, Neil M Walker, Jane Elliott

**Affiliations:** 1 Department of Health Sciences, University of Leicester, Adrian Building, University Road, LE1 7RH Leicester, UK; 2 JDRF/Wellcome Trust Diabetes and Inflammation Laboratory, Department of Medical Genetics, NIHR Cambridge Biomedical Research Centre, Cambridge Institute for Medical Research, University of Cambridge, Cambridge, UK; 3 Director of Cohorts and Longitudinal Studies Enhancement Resources (CLOSER), Centre for Longitudinal Studies, Institute of Education, London, UK

**Keywords:** Individual genetic research findings, Longitudinal population cohort, Ethics, Policy

## Abstract

Population-based, prospective longitudinal cohort studies are considering the issues surrounding returning findings to individuals as a result of genomic and other medical research studies. While guidance is being developed for clinical settings, the process is less clear for those conducting longitudinal research. This paper discusses work conducted on behalf of The UK Cohort and Longitudinal Study Enhancement Resource programme (CLOSER) to examine consent requirements, process considerations and specific examples of potential findings in the context of the 1958 British Birth cohort. Beyond deciding which findings to return, there are questions of whether re-consent is needed and the possible impact on the study, how the feedback process will be managed, and what resources are needed to support that process. Recommendations are made for actions a cohort study should consider taking when making vital decisions regarding returning findings. Any decisions need to be context-specific, arrived at transparently, communicated clearly, and in the best interests of both the participants and the study.

## Introduction

Whether and how to return ‘findings’ to individuals as a result of genomic and other medical research studies is being examined by population-based, prospective longitudinal cohort (hereafter ‘cohort’) studies. Many cohorts are now preparing DNA samples and collecting genetic data as part of their protocol and making these resources available to the research community [1]. Researchers accessing these samples and data are commonly conducting whole exome sequencing and whole genome SNP genotyping and analysis and finding information that might or might not have been a part of their original investigation. As the number of genomic resources increases, there is every expectation that the number of individual genomic research findings (hereafter ‘findings’) discovered will also increase [2,3].

There has been considerable debate surrounding the return of findings, but it is generally agreed that findings meeting the requirements of analytic validity, clinical significance and actionability should be considered for return. Other considerations include whether the individual has consented to receive findings, whether the findings are confirmed independently, and the seriousness of the condition indicated and the possibility that the intervention will improve the person’s health or lifestyle [4]. Findings that are not likely to benefit the individual should not be returned. In response, experts are working to develop practical guidance, for example, the American Council of Medical Genetics and Genomics (ACMG) recommendations address the feeding back of secondary findings from clinical whole-exome and whole-genome sequencing studies [5]. Their list of variants that should be returned has helped crystallise thinking, but in this rapidly changing area of science, lists such as these need to be fluid. Mutations will be found for new conditions, added to those already implicated in disease or found to not be the driver previously thought. As well, while markers, such as blood pressure levels will be different at various times, a person’s genotype is static and predictive throughout life. But even though someone’s genes may not change, there may be new signals, environmental influences or epigenetic conditions that will influence how a mutation will manifest itself phenotypically [6]. Therefore, even though a mutation is present, a person may not suffer from the condition or suffer in the same way or at the same point in life as others. This is a special burden on the provision of findings – balancing the knowledge that there is a moral imperative to give back findings that can be beneficial to individuals with the difficulty in having sufficient or timely information, such as validated data or consent, on which to base a decision that will convey that benefit.

The ACMG recommendations exclude research studies. Similarly, the UK10K project management framework outlines a process through which findings can be returned to participants in their study of the role low-frequency and rare genetic variants, but excludes those participants from population cohorts [7]. They acknowledge that their framework “…does not address the important issue of Ifs in population-based collections” [7]. This points to the fact that the feedback of findings in a clinical setting is very different from that for a research-focussed cohort. An individual’s health and well-being may be significantly improved by receiving findings, and this supports the argument for providing these to research participants. Returning findings can show respect to participants and demonstrate reciprocity for their participation [8]. However, while clinical studies can create generalizable research results as well as provide care for patients, the goal of research cohorts to build and maintain a resource for future research investigations.

Many long-standing cohorts that have a ‘no returns’ policy are now re-examining their position on returning findings [9]. The UK Cohort and Longitudinal Study Enhancement Resource programme (CLOSER) comprises nine studies: the MRC National Survey of Health and Development or 1946 cohort, the National Child Development Study or 1958 British Birth cohort (1958BC), the 1970 British Birth cohort, Understanding Society, the Hertfordshire Cohort Study, the Southampton Women’s Survey, the Millennium cohort, Avon Longitudinal Study of Parents and Children and the Life Study. All of these cohorts recruit ‘healthy’ volunteers, whose samples and associated data form a resource for future specified and unspecified research. With an increasing probability of discovering clinically significant findings at an individual level, CLOSER recognised the need to examine the latest evidence and guidance that could help their individual studies to form future policy on this issue.

We suggest that there are two main sets of decisions that need to be made by the leaders of cohort studies when examining this issue. First, whether it is ever appropriate to feed back findings. Second, if it is decided that individual-level findings could or should be returned in some circumstances, an appropriate feedback process needs to be chosen. This paper presents existing guidance available to cohorts, examines the process issues, and suggests a framework to assist cohorts to make informed policy choices.

### Analysis

As part of the infrastructure development and support programme 58READIE (Realising Easy Access to Data and Infrastructural Enhancement) of 1958BC, one of us (SEW) was asked to examine the pertinent issues to help inform future best practice. The 1958BC was chosen as an example of a cohort that was reconsidering its existing policy in the light of advances in genetic research. Members of this cohort were born in Scotland, England, and Wales during one week in 1958. The study has its origins in the Perinatal Mortality Survey, but over the subsequent nine surveys of members, or ‘sweeps’, the study has collected a broad range of data in a number of different domains including physical and mental health, health behaviours, education, employment, fertility, finances, social participation, values and attitudes.

In the 2002/3, 1958BC conducted a biomedical sweep, where samples of blood and saliva and associated data were collected from approximately 9,000 cohort members. This data forms part of the widely-accessed Wellcome Trust Case Control Consortium [10]. In order to examine access to 1958BC DNA, one of us (NW) compiled a list of approved applications. Some projects had found rare variants and there was the potential for findings that might be considered for return. This provided an opportunity to examine specific findings and the implications if they were fed back to participants. This led to a targeted examination of the current position of the 1958BC, the current UK policy landscape, and the implications and considerations for a possible change in policy.

Examples from the approved applications list were used to forecast the kinds of findings that the 1958BC might need to consider returning. Based on these, we reviewed the scientific literature to determine the implications disclosing this information to individuals. Only findings pertinent to 1958BC members (now aged 56) were considered. Other kinds of findings were excluded, such as those from MRI scans which are discussed elsewhere [11]. The existing consent materials from the 1958BC were examined to confirm current policy. To set this policy in context and to confirm current practice, we conducted a review for policies and guidance specific to the disclosure of findings, as well as the ethical debates, which a cohort study in the UK could use to inform its deliberations regarding a policy change. Only those written or translated in English and developed between 1995 and 2014 were considered. A briefing paper was presented and discussed at a CLOSER meeting in July 2012 [12].

### Existing policy and guidance on feeding back individual level genetic findings in cohorts

There is currently little guidance for researchers conducting longitudinal research, but some resources are now becoming available from funders and expert groups. In the UK, the Wellcome Trust and the Medical Research Council, in their *Framework for the return of health-related findings*, detail best practices and present case studies to suggest approaches researchers use to craft an appropriate feedback policy [13]. They recommend that findings be fed back when the potential benefits to an individual outweigh the potential harms, and if it is feasible to do so. Where findings will be fed back researchers should, “…develop a practical feedback pathway that is adequately resourced” [13]. If findings will not be returned, the reasons need to be clearly detailed and justified.

The Public Population Project in Genomics and Society (P^3^G) considered the return of varying categories of findings specifically in the context of population-based studies [1]. For these studies, traditionally, there has been feedback of results from assessments taken at the time of recruitment and storage of samples, and of general research results in the form of newsletters or websites [14]. The P^3^G statement stresses that any decisions regarding returning individual findings must be made in the specific context of each individual study and that, for some population-based studies, not returning findings, “…remains a viable option where appropriate” [1]. If an existing ‘no return’ policy is to be changed, the possibility of returning findings could be raised with participants if they are being re-contacted, perhaps for consent to participate in further research studies.

While the P^3^G statement addresses this issue at a general or conceptual level, others have examined the practical issues [15]. Implementation issues need to be considered, such as the need to re-confirm findings independently to ensure their accuracy, as well as to confirm the identity of the participant; legal responsibilities of secondary researchers, as well as the resource managers; and practicability and cost implications. Bledsoe *et al*. stress that each biobank needs its own policies and suggest that, if appropriate, an “…ethically defensible plan…” should be submitted to their ethics committee [15]. They advocate the position that “…return of individual research findings be considered on a case-by-case basis based on an evaluation of the risks and benefits to individuals and costs to society” [15].

### The salience of original consent and possibilities for re-consent?

The consent materials for the genetic component of the 2002/3 biomedical sweep of the 1958BC stated that ‘no information in the DNA will be given to [the participant].’ However, results from tests and measurements taken at the time of assessment, such as blood pressure and cholesterol readings, were reported to participants via their general practitioners (GPs). This now raises three possibilities – continuing with a policy of non-return of findings in line with original consents, returning findings on an ad-hoc basis in contradiction to original consent, or re-consenting those cohort members who had originally provided blood samples for DNA extraction. Experts recommend that participants be re-consented, governance-structure permitting, “…when the proposal deviates significantly from what was stated in the initial consent” [16].

The essential question is whether re-consent is a viable option. Evidence shows that individuals, when asked, say they want to have the choice to say yes or no to receiving findings [17], and by going through a re-consent process, researchers are acting transparently and showing respect for that participant’s contribution to the research [18]. However, re-consenting participants to seek a new use of their samples and data can have an impact on a cohort, as opposed to re-contacting them for purposes within the existing consent such as updating participant information. Participants may be unwilling to consent to the new terms and may withdraw. In practical terms, it may well not be possible to locate and contact all of the respondents, particularly when the study took place many years ago, again reducing numbers. Also, those who agree could be materially different than those who do not, possibly introducing selection bias due to over specific consent provisions (e.g. requirements such as the use of smartphone devices to participate that might only appeal to a certain segment of your population) and potentially affect the representativeness of results using the cohort [19]. Little work has been done on the impact of re-consent on cohorts, but evidence has shown that re-consent rates can be high. The international MONICA (Monitoring trends and Determinants in Cardiovascular Disease) project, in 2001, sought permission to use existing blood samples for ‘academic’ genetic research. The response was good; 93% of the 1409 participants agreed. However, the process itself still cost the project 13% of its participants through non-response and refusals [20]. A decision to try to re-consent study participants would therefore not be taken lightly and studies often seek the advice of an ethics committee [21].

### Which findings?

If a study decides that some individual-level findings could or should be fed back to participants, a vital issue becomes which ones should be returned. Even with existing guidance, it can still be difficult to specify what it actually means to meet the requirements for clinical significance and actionability. It can be difficult to develop guidelines which will be enduring because genetic technologies and scientific knowledge are continually developing. We discuss examples appropriate to the 1958BC and whether they would fit the criteria for feedback *if appropriate consents were in place*.

#### Findings already in clinical practice that should be returned

Variants in genes or loci that have shown analytical validity, clinical significance; and actionability should be considered for return to individuals. One example, cited by the ACMG and others [5,22], is familial hypercholesterolaemia (FH) which may be of relevance for studies such as the 1958BC with members over the age of 45. FH is a monogenic disorder that carries a high risk of premature coronary heart disease (>50% risk in men by age 50 and >30% in women by age 60); the estimated prevalence in the UK population is 1 in 500 [23]. The disease is treatable by statins and lifestyle changes, but is currently under diagnosed. UK NICE guidelines recommend against population screening, but that a suspected case of FH should be confirmed through genetic testing to confirm the diagnosis and to trigger cascade testing of relatives [23]. One expert (E. Birney, personal communication) has estimated that in a cohort such as the 1958BC of approximately 10,000, approximately 50 people will show a potentially actionable mutation predisposing heart attack through exome sequencing; a majority of these will be FH. Approximately 30-40 of these individuals will have their diagnosis confirmed, triggering cascade testing leading to 100-150 others to be approached regarding treatment. Members of the 1958BC who have consented will have had their cholesterol levels measured during the 2002/3 biomedical sweep. It is unknown how many 1958BC members are actually predisposed, but based on the above calculations, for a cohort its size, approximately 37 people could have a relevant mutation (again with the majority being FH), 20-30 of whom would have their diagnosis confirmed, with 75-100 further family members to be tested (i.e. through cascade testing). While there are currently no plans to exome sequence the 1958BC, many SNPs in the exome, including some of those targeted in FH testing, will have been measured as part of the current UK exome chip consortium and await analysis.

This argues for providing this information to those 1958BC members who have the mutation as there are existing clinical services available and should not require additional resources from the cohort. But, as has been argued (E. Birney, personal communication), members of the 1958BC may have already been diagnosed through regular clinical procedures. Additionally, if indicators of this disease were discovered at assessment for the biomedical sweep, individuals would have been advised to seek confirmation from a medical practitioner. But not everyone with the genetic mutation will suffer from the condition. Is the benefit to a relatively few individuals of knowing their FH status, which might be found otherwise, sufficient to impose a change of policy and the potential impacts of that change?

#### The unclear status of rare and preliminary findings

It is less easy to decide whether other classes of findings should be given back, such as rare mutations and widely available preliminary findings. One example studied in the 1958BC is the *NC_012920.1* mutation (also known as the *m.1555A > G* test), which can predispose individuals to be hypersensitive to aminoglycosides [24], used to treat infections such as multi-resistant strains of *Escherichia coli*[25]. Treatment with drug levels in the therapeutic range can cause profound and permanent deafness for individuals with this mutation. It may also cause late-onset hearing loss even in those not exposed to aminoglycosides. One in 400 of the 1958BC members tested showed the mutation and there was no evidence of hearing loss in middle age in the 1958BC [24]. However, Rahman *et al*., after studying the 1958BC, recommended that while population-level screening is not indicated, patients should have genetic testing for *NC_012920.1* prior to aminoglycoside administration in order to prevent inevitable hearing loss.

One can see potential benefit in returning this information. If a 1958BC member knew of their mutation they could request confirmatory genetic testing prior to administration or simply request that they not be given aminoglycosides. But the actual number of people with the *NC_012920.1* variant is small and aminoglycosides are currently not widely used, except in acute settings. Providing this information to people could cause them to worry about a situation that they may never face. Alternatively, individuals could suffer severe medical complications if such as finding is not given to them. Because of the potential for distress in either case, careful consideration would needed on whether, and how, to give this information.

Widely available preliminary findings, such as for coeliac disease, would most likely fall into the category of those findings that are low risk with clinical validity, and might be of some use to some individuals [22]. There is doubt regarding the precise phenotype for this condition and more work is needed to determine exactly which genes are causal [26]. But cohort members might want this information, as changes in lifestyle can improve the quality of life for sufferers. The cohort would need to weigh up the costs and benefits of providing individual feedback on these findings in this category.

### Infrastructure considerations

The return of individual-level findings is a complex issue with the potential to dramatically impact an individual’s life and any processes need to be well-considered to be beneficial for those involved. If at least some findings are to be returned, among the many things a cohort will need to consider is how to decide on which findings to return, how those findings will be returned, for how long the cohort will take responsibility for returning findings, and how these processes will be monitored and evaluated. Crafting a sensitive and appropriate process will show respect for the participation of cohort members, as well as for the cohort as a scientific study.

Reaching a consensus on which findings to feedback has been shown to be a difficult task as opinions differ and evidence may be lacking [2,27]. Table 1 shows some of the various options cohorts might have to assist them. It has been suggested that cohorts only give back findings that are already being treated in clinical practice [11]. Outside experts could be approached [28], or cohorts might join together to share common practice. In this latter case, studies need to be very similar, so that knowledge can be shared effectively. Existing resources are available to assist, such as the variants listed in the ACMG recommendations [5] or those from publically-available online resources, such as The Personal Genome Project GET-Evidence system or the Clinical Genomic Database [29,30]. Time and expertise will be needed to ensure that any list meets the needs of the cohort and is not simply a way to show that a response has been made to this important issue. As time passes and the science changes, cohorts will have the opportunity to monitor how their process is working. The input and experiences of participants will help to inform and potentially modify how approaches are taken. Reporting on these deliberations could add to the empirical evidence which will help others. Decisions on how long feedback of findings will continue, such as only for the lifetime of the study, will need to be taken and the reasons and implications discussed with participants.

**Table 1:** Options for routes through which cohort can make policy decisions on which findings to return

**Options**	**Advantages**	**Disadvantages**
Only return findings currently used in clinical practice	Clear utility for participants; no need for additional decision making body	Need to keep abreast of changes in clinical practice
Create own expert committee to decide upon which findings to return	Study-specific; greater control over decision making process	Potentially resource intensive (i.e., funds to staff/maintain committee, time donated by members)
Join other cohorts to form a joint committee	Greater combined expertise; shared costs	If studies are not similar, decisions may be divisive or not useful
Rely on the committee of another cohort	Inexpensive; no need for additional decision making body	Decisions may not be specific enough for own cohort; no power to oversee/direct decision making process
Rely on resources (i.e., lists or databases) created by other expert groups	Based on the latest evidence; freely-available resources now available	Data may not be specific enough or appropriate for cohort; if fee-based, could be expensive; need time/expertise to apply their data to own cohort

When secondary researchers outside the cohort report findings, the important issues of communication and validation need to be considered. Losing information, giving incorrect information, or withholding correct information, could have serious implications for all those involved [7,31]. To confirm findings, secondary researchers could be asked to re-analyse materials, validation be done in-house by the cohort, or outsourced to an independent laboratory for a second opinion using a DNA sample from the same participant if available [32]. Appropriate measures will help to avoid the potential of causing unnecessary distress through poor management. As with participants, secondary researchers will also need to be clear as to how long they will be responsible for returning findings. They might expect their responsibilities to end at the conclusion of their own funded study and agreed use of the cohort data, yet the cohort study will most likely be still in place. If the cohort has clear management procedures for receiving and returning findings, this should enable secondary researchers to be in contact with information when necessary.

Respecting an individual’s right to know and their right not to know are important ways of showing respect for autonomous decision making. Through a re-consent exercise or in prospective consent materials for a new study, participants will be able to record whether or not they wish to be re-contacted with findings. Any decision on overriding that decision needs to be based on whether the benefit to the individual could outweigh their potential distress in learning the information. Advice from an oversight committee, such as a research ethics committee can help with such decisions [1].

Finally, participants will need help to cope, both clinically and emotionally, with any findings given to them. This is of course particularly important in relation to genetic findings that may have implications for other members of the family. In a recent study, participants said they preferred that health-related findings be given to them in a face-to-face setting, and “…generally…from someone with medical knowledge and expertise, who could ensure the finding was followed-up appropriately: usually a GP or a specialist healthcare professional” [17]. The Wellcome Trust/MRC Framework gives an example of having a clinical geneticist on the cohort research team who could review potential cases and deliver the feedback; this would help participants adjust from being a ‘healthy volunteer’ to someone in need of clinical care. Alternatively, information could be passed directly to the participant’s GP who would then contact them for a consultation. This presupposes cohort members have a relatively stable, on-going relationship with a GP or practice which will not be the case for all. For whoever delivers the findings, making the information ‘understandable’ is key, as explaining genetic risk to lay persons can present difficulties [33] and it is vital that additional distress is not caused due to clumsy communication. Wolf *et al*. recommend that “[f]indings should be returned in a form that is understandable to the [participant] and useful to a physician or other clinician, such as a genetic counsellor…” [4].

It is impossible to estimate how many findings will be discovered over the life of a cohort project such as the 1958BC, but regardless of the number, ensuring that there is adequate funding to maintain the system will help to ensure that individuals are given information in a timely and appropriate manner. The costs related to the return of findings and the subsequent care involved, and how those costs will be covered will differ from country to country and setting to setting, but limited evidence shows that returning findings can be expensive [34,35]. Secondary researchers could help with planning by informing the cohort if they expect to obtain findings of potential clinical significance to individuals. Some funders are now asking for this information as part of protocols [36,37]. Also, some costs could possibly be passed on to the researchers in order to help fund the process, perhaps through fees included in data access agreements.

### Is there an argument for not returning findings to cohort members?

It has been suggested that population-based studies can decide to not return findings [1]. This would help preserve the distinction between research and healthcare [38]. By returning findings, cohort research may begin to resemble screening, but without the rigorous guidelines designed to protect individuals. In addition, there could be a cost to society through shifting resources from research to individual care [15]. However, there is an argument that the positive impact on one person’s life through the feedback of a finding should outweigh any administrative costs or loss of membership for a cohort, leading to finding new ways in which to balance the potential benefits to individuals with the nature of longitudinal research. Dynamic consent models, for example, allow participants to state preferences for feedback [39], although it has been argued that these could contribute to the conflation of healthcare and research in the minds of participants [40].

For some studies there is one clear way to justify a decision to not return findings – by ensuring that there will be no findings to return. This can be done by refusing to grant access for any analysis (most likely genetic) that might produce clinically significant findings. This would preserve studies like the 1958BC that began before the genomic revolution and may have only added genetic studies recently. It is questionable whether limiting potential research uses is ethically-defensible. But equally, it is questionable whether a cohort successfully supplying data for a wide range of research (i.e., educational, social science and epidemiological research in the case of the 1958BC) should be potentially damaged for the sake of genetics.

Given that the original consents with members of the 1958BC explicitly stated that findings would not be returned to cohort members, the team responsible for this cohort has decided that individual findings will not currently be returned. This will remain under review. The team recognises that consents written a half a decade ago could not foresee the use of genomic analysis in individual care. The next face to-face survey of the cohort is planned for 2018, when cohort members are aged 60, and an important part of the planning of this data collection will be the decision about whether to re-consent study members so that findings can be returned in future. Indeed it may well be that it is decided to re-collect DNA via saliva or blood at that time. The considerations discussed in this paper, future guidance and evidence from other cohorts will be very important for informing the process of obtaining informed consent that enables cohort members to decide whether they wish to receive feedback on any findings, and will also inform the structures and processes that would need to be funded and put in place to support any return of findings.

## Conclusion

It is clear that people report that they want findings, “…when a condition is serious and treatable”, [17] and want to choose what findings to receive and how frequently [41]. Whether these desires will in the future become demands, potentially affecting recruitment and retention, is unknown. Any decisions made by a cohort will need to be context-specific, arrived at transparently and justifiable to its members. In particular, each cohort will need to:

•Examine their consent provisions and decide if consent is in place to return findings

•If consent is not in place, decide if it is necessary, feasible, and in the best interests of scientific mission of the cohort and of its members, to go through a re-consent procedure; and if so, when.

•If findings are to returned, decide how the variants to be returned will be chosen, how they will be validated, confirmed and given to individuals.

•Write, and publically disseminate, a policy statement, reflecting national and international best practice guidance, that includes a list of the genetic variants and how they were chosen (by whom and using what criteria); the process that will be used to return findings; and the responsibilities of the cohort and of secondary researchers.

•Agree on an on-going monitoring programme, including a periodic review of the list of variants, whether some findings are appearing more frequently than others, and whether the feedback process should be updated or changed to ensure effectiveness.

•Ask cohort members, whether as research or consultation, how the processes are working for them and use that information to inform the on-going review of policies and procedures.

Only through a transparent evidence-based examination of the issues will cohort study managers be able to balance the needs of their members with the scientific integrity of the cohort, in order to ensure the best interests of both.

## Competing interests

The authors declare that they have no competing interests.

## Authors’ contributions

SW designed and coordinated the study, conducted the analyses and drafted the manuscript. NW provided data, participated in the design of the study and assisted with analyses. JE participated in its design and coordination and helped to draft the manuscript. All authors read and approved the final manuscript.
